# Transcriptomic plasticity of cholinergic adipose macrophages in the acute thermogenic response

**DOI:** 10.1016/j.jbc.2025.110925

**Published:** 2025-11-07

**Authors:** Alexander J. Knights, Evan J. Kim, Shanshan Liu, Jun Wu

**Affiliations:** 1 Life Sciences Institute, University of Michigan, Ann Arbor, Michigan, USA; 2 Department of Orthopaedic Surgery, Washington University, St Louis, Missouri, USA; 3 Center of Regenerative Medicine, Washington University, St Louis, Missouri, USA; 4 Department of Molecular & Integrative Physiology, University of Michigan, Ann Arbor, Michigan, USA

**Keywords:** adipose tissue, macrophage, acetylcholine, transcriptomics, single-cell RNA-seq

## Abstract

Cholinergic adipose macrophages (ChAMs) have recently been shown to regulate the acute thermogenic response in subcutaneous white adipose tissue, yet their transcriptomic dynamics are poorly understood, and little is known about their origins or identity. Using single-cell RNA sequencing (scRNAseq), we profiled ChAT-eGFP+ cells (expressing choline acetyltransferase) from subcutaneous white adipose tissue of mice housed at thermoneutrality or after acute cold exposure. We identified twelve distinct clusters of ChAT-expressing cells, predominated by hematopoietic cell types. Specifically, ChAMs exhibited increased proportions and *Chat* expression after acute cold. Widespread differential gene expression was induced in ChAMs after cold compared to thermoneutrality, with cold-enriched pathways in immune signaling, chemotaxis, and metabolism. Several ChAM subsets were uncovered that resembled previously reported adipose macrophage subpopulations. ChAMs were predicted to have mixed origins, derived from adult bone marrow and embryonically. These findings provide a high granularity assessment of cholinergic immune cells in fat, and we highlight the transcriptomic plasticity and mixed origins of ChAMs, suggesting their therapeutic potential for metabolic diseases.

The significant influence that adipose tissue has on systemic energy balance and whole-body metabolic health has long been recognized. Adaptive thermogenesis in adipocytes can be considered a “fight or flight” response towards environmental challenges such as cold exposure, and sympathetic (adrenergic) regulation of this process has been the subject of numerous investigations (1). However, this paradigm has broadened since the identification of cholinergic receptor nicotinic alpha two subunit (CHRNA2) as a key regulator of thermogenic beige adipocyte function in both mice and humans (2, 3, 4, 5). The broad consensus within the adipose field is that there is no significant presence of parasympathetic innervation within subcutaneous adipose tissue (6), where beige adipocytes are prominent. Recently, it was revealed that acetylcholine derived from immune cells expressing choline acetyltransferase (ChAT) within subcutaneous adipose tissue regulates CHRNA2-dependent signaling in activated beige adipocytes *via* paracrine crosstalk (2, 7). In particular, cholinergic adipose macrophages (ChAMs) have been shown to regulate the thermogenic response to acute cold challenge (7).

As evidenced by these intricate crosstalk interactions during thermogenic activation, adipose tissue is a dynamic organ, home to diverse cell types and highly responsive to internal and external cues (8, 9). Amongst the plethora of cell types that influence fat function, immune cells such as macrophages have been extensively investigated for their essential regulatory role in controlling fat activity and the thermogenic response (10, 11). Up until recently, single-cell analyses such as flow cytometry were the main approaches underpinning our knowledge of cellular identities and diversity in tissues like fat, but despite progress in multiparametric analysis, these approaches suffered from low dimensionality. However, more recent advances in single-cell and -nucleus RNA-sequencing technologies have allowed for whole-transcriptome profiling and brought paradigm-shifting changes to our understanding of the complex nature of adipose tissue composition and molecular patterns in metabolic normalcy and dysfunction (12, 13, 14, 15).

Here, we used single-cell RNA-sequencing (scRNAseq) to investigate the cellular and transcriptomic signatures and dynamics of cholinergic immune cells from subcutaneous adipose tissue of mice housed at either thermoneutrality (30 °C) or after thermogenic activation by acute cold exposure (4 °C).

## Results

### Identification of ChAT-expressing cell types by gene signature

**Figure 1:**
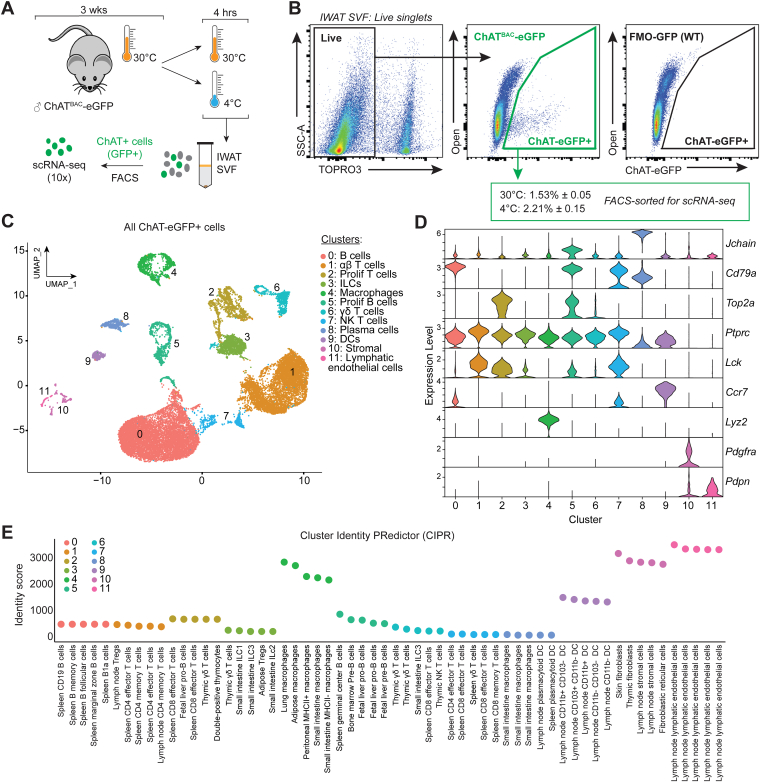
**Identification of ChAT-expressing cell types by gene signature.***A*, schematic showing the experimental workflow (n = 2 biological replicates per condition). Mice were housed at thermoneutrality (30 °C) then acutely challenged with cold (4 °C) or kept at thermoneutrality for 4 h, prior to FACS sorting of ChAT-eGFP+ cells from inguinal white adipose tissue stromal vascular fraction (IWAT SVF). *B*, FACS gating of live, single ChAT-eGFP+ cells. Cells from wild-type (WT) mice were used as a fluorescence-minus-one (FMO) control for GFP. Mean proportion of ChAT-eGFP+ cells as a percentage of all live single cells is given ± SEM. *C*, UMAP plot showing 12 clusters (0–11) of ChAT-eGFP+ cells from IWAT SVF; both conditions merged. Prolif: proliferating; ILCs: innate lymphoid cells; NK T: natural killer T cells; DCs: dendritic cells. *D*, Violin plots showing expression of cluster-defining marker genes. *E*, output from cluster identity predictor (CIPR) program showing the top five predicted cell types for each cluster based on gene signatures from RNA-seq of FACS-sorted murine cell types.

Previously we characterized ChAT-expressing cells in adipose tissue by flow cytometry using ChAT^BAC^-eGFP mice (2, 7). For a more granular characterization of adipose cholinergic cells, based on their transcriptomic signature, we performed single-cell RNA-sequencing (scRNAseq) on ChAT-eGFP+ cells from the stromal vascular fraction (SVF) of subcutaneous inguinal white adipose tissue (IWAT). ChAT-eGFP mice were housed at thermoneutrality (30 °C) for 3 weeks then given an acute cold challenge at 4 °C for 4 h, or kept at thermoneutrality (Fig. 1*A*). IWAT was harvested and digested to yield the SVF, from which ChAT-eGFP+ cells were sorted by FACS (Fig. 1*B*) and prepared for 3′ GEX scRNAseq. Cell viability for sorting was ∼80% and, in line with our previous findings using room temperature as a baseline (2, 7), acute cold exposure increased the proportion of ChAT-eGFP+ cells compared to thermoneutrality (Fig. S1, *A* and *B*). Following rigorous quality control and filtering of sequencing data, a total of 10,579 cells and 10,674 cells, from 30 °C and from 4 °C respectively, were used for downstream scRNAseq analyses (Fig. S1*C*). ChAT+ cell types were visualized using UMAP dimensionality reduction agnostic to condition (Fig. 1*C*), revealing 12 distinct clusters (0–11) based on unique gene expression signatures. The identities of these clusters were determined using unbiased cell cycle phase analysis, targeted gene marker analysis, and the use of a cluster identity prediction tool called CIPR (16) (Figs. 1, *C*–*E* and S1, *D*, *E*, and Table S1). We detected B cells (cluster 0) and plasma cells (cluster 8), αβT cells (cluster 1) and ɣδT cells (cluster 6), innate lymphoid cells (ILCs, cluster 3), macrophages (cluster 4) and dendritic cells (DCs, cluster 9), natural killer T cells (NK T cells, cluster 7), proliferating T cells (cluster 2) and B cells (cluster 5), and small populations of stromal (cluster 10) and lymphatic endothelial (cluster 11) cells. These cell populations broadly corroborate those we originally characterized using flow cytometry (2, 7) but with greatly increased resolution and the ability to assess global gene expression, functions, differentiation, and activation dynamics on a per-cell basis.

### Cholinergic adipose macrophages are responsive to acute cold challenge

**Figure 2:**
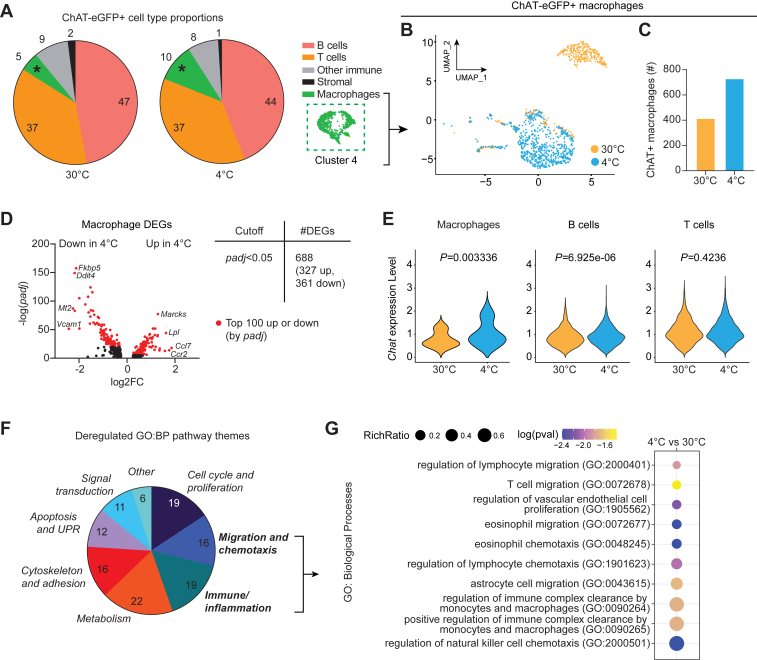
**Cholinergic adipose macrophages are responsive to acute cold challenge.***A*, pie charts showing the proportional breakdown of broad ChAT-eGFP+ cell populations at 30 °C and 4 °C. Percentages are given for each population. *Asterisk* highlights the macrophage (*green*) proportional increase at 4 °C. *B*, macrophages (cluster 4) were computationally subset and re-clustered using Seurat then projected onto a UMAP and colored by condition. *C*, total abundance of ChAT-eGFP+ macrophages at 30 °C and 4 °C. *D*, Volcano plot and corresponding table showing all differentially expressed genes (DEGs) between macrophages from 30 °C and 4 °C with a cutoff of *padj*<0.05. Genes in *red* represent the top 100 up or down DEGs (ranked by *padj*). *E Chat* expression in macrophages (*left*), B cells (*middle*), or T cells (*right*) at 30 °C and 4 °C, with statistical significance (*p* value) assessed by Wilcoxon rank sum testing. *F* The top 100 up- and down-regulated DEGs from *D* were used as input for pathway analysis for Gene Ontology: Biological Processes (GO:BP), and the broad themes of deregulated pathways are shown. The number of terms attributed to each theme are shown in each pie chart slice. *G* Bubble plot of GO:BP pathways enriched in macrophages from 4 °C derived from the ‘Migration and chemotaxis’ or ‘Immune/inflammation’ themes. RichRatio is a ratio of the number of DEGs found in a pathway term over the total number of genes in that term. Log(pval) indicates significance using the statistical enrichment test in PantherDB.

Following on from our detailed characterization of cholinergic cell types in IWAT, we sought to assess the effect of acute cold challenge on these cells. Since total cell abundance cannot be directly assessed in scRNAseq when the number of input cells per condition is equalized, we instead analyzed cell type proportions between conditions. The proportional breakdown of broad ChAT+ cell populations closely resembled our original findings (2, 7) (Figs. 2*A* and S2*A*). Extending on these original findings, we showed by flow cytometry that ChAT-eGFP+ immune cells were elevated in acute cold exposure compared to room temperature and thermoneutrality (Fig. S2*B*). The scRNAseq data showed significant *Chat* transcript elevation in cells at 4 °C compared to 30 °C, and overall cholinergic signaling was increased based on composite expression of genes in the acetylcholine pathway (Fig. S2, *C* and *D*). Of note, our scRNAseq data provided additional support for the observation that cholinergic macrophages (ChAMs, cluster 4) proportionally increase relative to other cholinergic cell types in IWAT following cold exposure (7) – this time with thermoneutrality as the baseline control (Fig. 2, *A*–*C*). Cells in the macrophage cluster were computationally isolated for more targeted analyses without confounding noise from other ChAT+ cell types. Pairwise differential gene expression analysis revealed significant transcriptional changes in ChAMs after cold exposure compared to thermoneutrality, with 688 differentially expressed genes (DEGs) using a *padj*<0.05 cutoff (Fig. 2*D* and Table S2). Among these, *Chat* transcript itself was significantly up-regulated in cold ChAMs (Fig. 2*E*), corroborating prior results showing elevated ChAT-eGFP signal intensity following cold exposure (7). B cells also showed elevated *Chat* in cold, but T cells did not (Fig. 2*E*). *Adrb2* was up-regulated in cold ChAMs (Fig. S2*E*), relevant to our prior finding that β_2_-adrenergic signaling regulates ChAM function in thermogenic activation (7). Seeking to understand aggregate global changes beyond individual gene expression, we performed pathway analyses to identify enriched functions in ChAMs after cold exposure compared to thermoneutral housing. The top 100 up- and down-regulated DEGs, as ranked by *padj* value (Fig. 2*D* and Table S2), served as input for Gene Ontology (GO) and Reactome analyses using PantherDB (Figs. 2, *F*, *G* and S2, *F*, *G*). GO (Biological Process) analysis revealed deregulated functions covering various broad pathway themes, including signal transduction, migration and chemotaxis, immune and inflammation, metabolism, and cell proliferation. Closer interrogation of GO terms within the migration/chemotaxis and immune/inflammation themes revealed that, relative to thermoneutrality, ChAMs from 4 °C were enriched for pathways relating to trafficking of lymphocytes, NK cells, and eosinophils—all cell types that have been strongly linked to thermogenic activation (17, 18, 19, 20, 21, 22) —along with endothelial cell proliferation and monocyte/macrophage immune complex clearance (Fig. 2*G*). GO Molecular Function analysis revealed that cold-exposed ChAMs were enriched for chemokine signaling terms, ion binding, and cellular enzymatic activity (Fig. S2*F*) and Reactome analysis indicated that terms related to MHC class II presentation and heme scavenging were enriched in ChAMs after cold exposure (Fig. S2*G*). Together these data demonstrate the dramatic transcriptional changes that ChAMs undergo during acute cold exposure, and that these changes underlie enrichment of specific cellular functions related to inflammation, chemotaxis, cell signaling, and other broad functional domains, emphasizing the plasticity of these cells.

### Cholinergic adipose macrophages exhibit heterogeneous gene signatures

**Figure 3:**
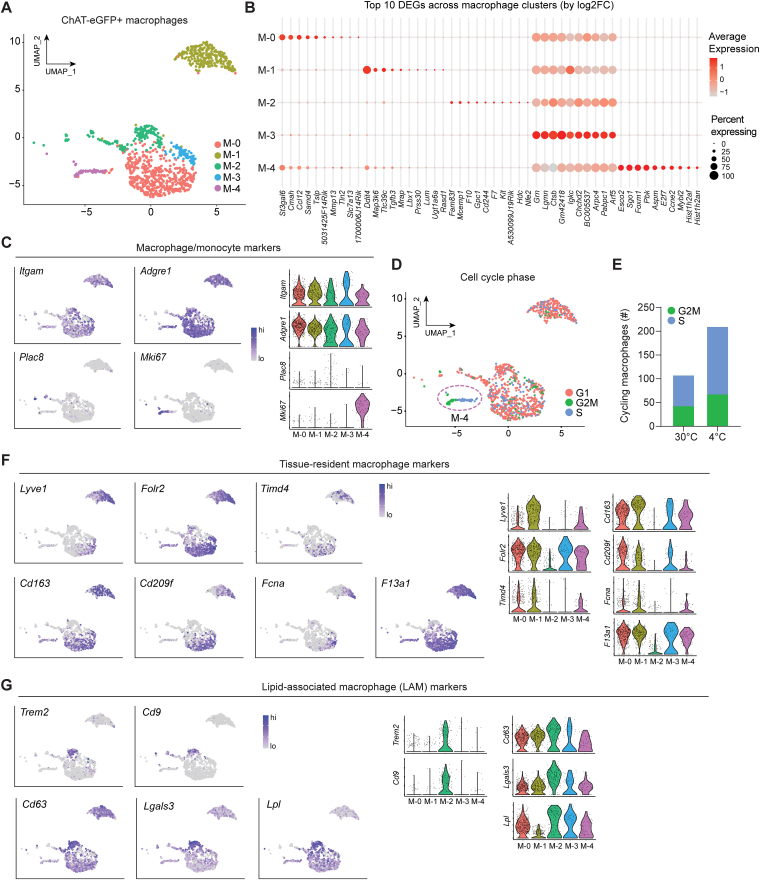
**Cholinergic adipose macrophages exhibit heterogeneous gene signatures.***A*, UMAP showing clustering of ChAT-eGFP+ macrophages. *B*, bubble plot showing top 10 DEGs by log2FC for each cluster (M-0, 1, 2, 3, 4). *C*, common macrophage/monocyte marker genes displayed as feature plots (*left*) or violin plots (*right*). *D*, UMAP of ChAT-eGFP+ macrophage clusters colored by cell cycle phase. Proliferating macrophages (S/G2M phases) are circled, and correspond to cluster M-4. *E* Total number of G2/M and S phase proliferating ChAT-eGFP+ macrophages in both conditions. *F*–*G* Expression of tissue-resident macrophage marker genes (*F*) and lipid-associated macrophage (LAM) genes (*G*), displayed as feature plots (*left*) or violin plots (*right*).

Since the advent of single-cell transcriptomics, we have been given unparalleled resolution into cellular heterogeneity; macrophages are no exception. Studies in the past decade have uncovered a plethora of macrophage functional subsets and phenotypes across tissues and disease states, including in fat (15, 23, 24, 25). We sought to determine whether acetylcholine-secreting macrophages represent their own distinct subset of macrophages or whether cholinergic identity and function can be assumed by macrophages of diverse functional phenotypes. Having computationally subset only macrophages (Fig. 2, *D* and *E*), semi-supervised clustering was performed and it uncovered five biologically meaningful and distinct subpopulations of ChAMs based on unique, differential gene signatures (M-0, 1, 2, 3, 4) (Fig. 3, *A* and *B*). Using targeted marker-based identification, we saw broad expression of canonical macrophage markers *Itgam* (encoding CD11b) and *Adgre1* (encoding F4/80) across ChAMs, with a small population expressing the monocyte marker *Plac8* and others expressing the proliferative marker *Mki67* (encoding Ki67) (Fig. 3*C*). Cell cycle phase analysis confirmed that *Mki67* expression was confined to a subset of proliferating cells (cluster M-4), split across both the S phase and G2/M phase (Fig. 3*D*). This points towards ChAMs being locally proliferative, in agreement with our previous data showing Ki67 positivity by flow cytometry (7). Of note, the number of proliferating ChAMs was increased in the cold, compared to thermoneutral (Figs. 3*E* and S3*A*). We then examined our data for expression patterns of genes previously reported to mark subsets of macrophages in other tissues and disease settings. Numerous papers have now defined hallmarks of tissue-resident macrophages (24, 25, 26, 27), and we detected canonical resident markers *Lyve1, Folr2, Timd4, Cd163, Cd209f, F13a1* and *Fcna* in our clusters M-0 and M-1, containing cells from cold and thermoneutral conditions respectively (Fig. 3*F*). It is worth noting that cluster M-1 (predicted to be tissue resident) was entirely comprised of cells from the thermoneutral condition, whereas cold-derived cells were more evenly dispersed between clusters predicted to tissue resident (M-0, M-4) and bone-marrow derived (M-2, M-3) (Figs. 2*B* and 3, *A*, *F*). Cluster M-2 bore hallmark genes of lipid-associated macrophages (LAMs), which have been reported in fat and other tissues and play a key role in obesity and lipid scavenging: *Trem2, Cd9, Cd63, Lpl* and *Lgals3* (28, 29, 30, 31, 32, 33, 34) (Fig. 3*G*). Interestingly, although the “M1-M2” macrophage polarization binary is outdated, we did observe non-overlapping expression of the classic “M1” marker *Itgax* (encoding CD11c) in cluster M-2 resembling LAMs, and the classic “M2” marker *Mrc1* (encoding CD206) in the resident macrophage clusters M-0 and M-1 (Fig. S3*B*). However, we did not detect appreciable expression of other classic M1/M2 markers, *Nos2* (encoding iNOS) or *Arg1* (encoding Arginase). Tissue macrophages associated with nerves (NAMs) have been discovered in fat and other tissues (35, 36, 37, 38) and interestingly, expression of key NAM genes such as *Maoa, St3gal6* and *Lilra5* was confined to our resident clusters M-0 and M-1 (Fig. S3*C*). Our data suggest that the clusters enriched for NAM genes (M-0 and M-1) are tissue-resident cells, given their expression of *Lyve1*, *Folr2* and *Timd4* (Fig. 3*F*). This aligns with their proposed resident status in other recently published reports (24, 37), and notably, while these cells are present in both temperature conditions, they are especially enriched in cold (cluster M-0) (Figs. 3*A* and S3*C*). Genes shown to have roles in macrophage-mediated inflammatory and metabolic modulation (39, 40, 41, 42, 43) were enriched in the M-1 resident cluster that is represented entirely by cells from the thermoneutral condition (Fig. S3*D*). Together, these data suggest that various tissue macrophage subsets possess the capacity to secrete acetylcholine and may be functional in thermal responses.

### Origins and differentiation of cholinergic adipose macrophages

**Figure 4:**
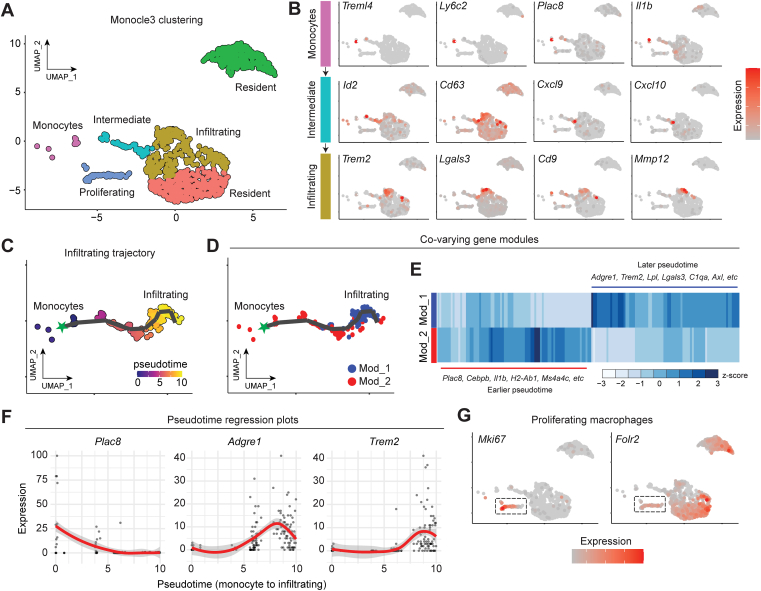
**Origins and differentiation of cholinergic adipose macrophages.***A*, UMAP showing reclustering of ChAT-eGFP+ macrophages in Monocle3, annotated with putative cluster names based on differential gene expression analysis. *B*, feature plots showing expression of genes confined to the monocyte (*top*), intermediate macrophage (*middle*), or infiltrating macrophage (*bottom*) clusters. *C* Pseudotime trajectory from monocytes (origin, *green* star) to infiltrating macrophages. *D*–*E* Modules of co-varying genes across pseudotime were calculated (Mod_1 and Mod_2) and projected onto the UMAP trajectory (*D*) or displayed as a differential gene expression heatmap (expression shown as normalized z-score) (*E*). Key module genes that co-vary with pseudotime are listed with the heatmap. *F*, Pseudotime regression plots showing expression of *Plac8* (monocyte marker), *Adgre1* (macrophage marker, F4/80), and *Trem2* (infiltrating macrophage/LAM marker) across the monocyte to infiltrating macrophages trajectory. *G*, Feature plots showing expression of proliferation marker *Mki67* and the tissue resident macrophage marker *Folr2* in the outlined proliferating cluster.

Fate mapping studies have shed light on the disparate origins of macrophages across tissues and disease states (30, 44, 45, 46). Our previous study indicated that ChAMs likely derive from both a bone marrow origin and from a local, self-renewing resident pool (7). The presence of ChAT-eGFP+ monocytes in our dataset (Fig. 3*C*), whether captured from the adipose vasculature or in the process of extravasating into the tissue, supports a population originating in bone marrow, while the presence of ChAT-eGFP+ macrophages expressing *Lyve1*, *Timd4* and *Folr2* point to a resident population (Fig. 3*F*). To study the origins and differentiation dynamics of ChAMs in more depth, we used the trajectory analysis package Monocle3. Monocle clustering revealed clearly defined resident macrophages and proliferating macrophages first shown in Figure 3 (Fig. 4*A*). In addition, we identified three clusters bearing hallmarks of monocytes that are recruited to fat from bone marrow and infiltrate into the tissue to become macrophages (Fig. 4*B*). We termed these clusters monocytes (*Treml4, Ly6c2, Plac8, Il1b*), intermediate macrophages (*Id2, Cd63, Cxcl9, Cxcl10*), and infiltrating macrophages (*Trem2, Lgals3, Cd9, Mmp12*), the latter of which align with the cells resembling LAMs (Fig. 3*G*), which are thought to be recruited from circulation (30). Semi-supervised trajectory analysis using the monocyte cluster as the origin showed these cells transitioning into intermediate then infiltrating macrophages across pseudotime (Figs. 4*C* and S4, *A*, *B*), correlating with increasing expression of the tissue macrophage marker *Adgre1* (F4/80) and greater overall per-cell transcript expression (Fig. S4, *C* and *D*). Co-varying gene modules across the monocyte-to-infiltrating macrophage trajectory were determined (Fig. 4, *D* and *E* and Table S3), revealing shifts in gene expression profile as ChAT-eGFP+ monocytes are recruited to adipose tissue, illustrated by pseudotime regression plots of *Plac8*, *Adgre1*, and *Trem2* expression (Fig. 4*F*). We then performed gene ontology analysis on pseudotime trajectory gene modules one and 2 (Fig. S4*E* and Table S4). Module 2, containing genes enriched earlier in pseudotime (monocytes), was heavily dominated by terms pertaining to inflammation and the immune response. However, the module one genes that were highly expressed later in pseudotime (recruited macrophages) were overrepresented in a diverse range of biological pathway themes pertaining to functions in metabolism, adhesion, apoptosis, inflammation, migration, and signal transduction. Of note, the proliferating cell cluster, marked by *Mki67*, bore expression of the resident macrophage marker *Folr2*, further supporting the presence of a local, cycling population of acetylcholine-synthesizing adipose-resident macrophages (Fig. 4*G*). Together, these data support the notion that ChAMs derive from mixed origins, opening the possibility that both local and systemic cues may govern the cholinergic identity and functions of macrophages.

## Discussion

In this study we performed an unbiased assessment of the transcriptomic landscape of cholinergic cells within inguinal subcutaneous fat, at thermoneutrality and after acute cold challenge. These scRNAseq data and our prior work (2, 7) have collectively demonstrated that ChAMs are dynamically regulated by cold exposure at the gene and functional level. In recent years, the functional significance of non-neuronal cholinergic regulation of bodily function has been increasingly appreciated (47, 48). In addition to thermogenic regulation *via* ChAMs, acetylcholine derived from assorted immune cells have been implicated in various physiological processes, including blood pressure regulation (49), anti-viral and anti-parasite responses (50, 51, 52, 53), and liver function (54, 55). Accumulating evidence supports a vital role for non-neuronal cholinergic signaling in human physiology (56, 57). It will be of particular significance to investigate whether ChAMs in humans share similar molecular signatures and are regulated *via* signaling pathways identified in our studies, when responding to changes in energy balance.

Prominent within subcutaneous adipose tissue, thermogenic beige adipocytes have been intensively studied under environmental and metabolic stresses, such as cold exposure, thermoneutrality and obesity. It has recently been shown that cholinergic regulation of beige adipocytes may also influence recovery and adipose tissue remodeling after burn-injury (58). Previous work on crown-like structures of adipose macrophages in obesity and their impact on fat dysfunction emphasizes that the spatial context of adipose macrophages could be key to their physiological consequences, in addition to their identity or abundance (33). Given the short half-life of acetylcholine *in vivo*, it is conceivable that ChAMs physically locate within close proximity to activated beige adipocytes, whose spatial distribution has already been investigated under various environmental and metabolic contexts (59). Future investigations focusing on the spatiotemporal organization of ChAMs in various metabolic contexts, such as obesity, cold challenge, or the post-burn hypermetabolic state, will reveal further insights into the function of these cholinergic myeloid cells in metabolic regulation.

The origins and identities of tissue macrophages are widely studied themes in immunology, including in an adipose context. Distinct adipose macrophage subtypes have been characterized, based on gene signature and function, including lipid-associated macrophages (LAMs) (29, 30), nerve-/sympathetic nerve-associated macrophages (NAMs/SAMs) (35, 36, 37), plus macrophages that resemble archetypal pro-inflammatory (“M1”) and anti-inflammatory (“M2”) cells. Likewise, fate mapping studies have provided valuable insight into the embryonic and adult origins of macrophages, and how this may influence their homeostatic and disease functions (24, 25, 30, 44, 45, 46). We uncovered hallmark gene signatures of various reported adipose macrophage subtypes in the ChAT-eGFP+ macrophage pool, including LAMs, NAMs/SAMs and M1/M2-like cells. This, coupled with our finding that ChAMs appear to arise from both a bone marrow origin (recruited) and an embryonic origin (tissue resident), suggests that cholinergic function in macrophages is an assumed function that may be highly dynamic and malleable, and thus potentially exciting for therapeutic intervention. This assertion is underscored by the dynamic changes that occur in ChAMs during cold exposure, when compared to ChAMs derived from mice housed at thermoneutrality, where there is no requirement for endogenous body heat generation and metabolic tissues like fat assume a more lipogenic phenotype. We found that the majority of ChAMs from mice housed at thermoneutrality were predicted, based on their transcriptional signature, to be an entirely tissue-resident population. However, after cold exposure, ChAMs occupied clusters representing both resident and bone marrow-derived origins, suggesting that acute cold exposure may stimulate influx and local activation of cells poised to assume cholinergic functions. The cold-induced resident population bore hallmarks genes expressed by NAMs that associate with nerves and regulate adipose homeostasis and age-induced inflammation (37). Our findings suggest that NAMs may be cold-responsive and have the capacity for acetylcholine synthesis to regulate fat function. Further underscoring this notion is the observation that ChAMs expressing NAM-associated genes were also found in the proliferating cluster, pointing towards a local, self-renewing population that is more prominent in cold exposure.

To extend upon the limitations of gene signature-based analyses, future investigations must incorporate rigorous functional evaluation and genetic lineage tracing of ChAMs, made possible by advances in the ability to distinguish between bone marrow-derived *versus* tissue resident origins (45, 60). Further, as newer, more specific cre drivers emerge for studying functional subsets of macrophages, we will be better positioned to dissect the protective *versus* pathological functions of ChAMs in different metabolic states. These efforts, combined with an understanding of the spatial niches in which ChAMs may reside, will shed important light on the role of these cells in metabolic regulation.

## Experimental procedures

### Mice

A schematic of the experimental design can be found in Figure 1*A*. At 5 weeks of age, male ChAT^BAC^-eGFP mice were housed at thermoneutrality (30 °C) for 3 weeks. Following this period, mice were either kept at thermoneutrality or exposed to an acute cold challenge at 4 °C for 4 h. Mice in the cold exposure groups were acclimated at room temperature for 24 h prior to cold challenge, to minimize the likelihood of attrition. Two biological replicates were performed per condition (thermoneutrality or cold exposure), with each replicate comprised of four pooled mice harvested at approximately 8 weeks of age. Mice were housed according to a 12-h light/dark cycle and given free access to water and chow feed. All animal studies were reviewed and approved by the Institutional Animal Care and Use Committee at the University of Michigan (protocol number PRO00012391).

### Harvest and sample preparation

Inguinal subcutaneous white adipose tissue (IWAT) was immediately harvested after CO_2_ euthanasia, finely minced, and then digested to yield the stromal vascular fraction (SVF). Digestion was performed as previously reported (7), in a Collagenase D solution (1.5 U/ml) and Dispase II (2.4 U/ml) supplemented with 10 mM CaCl_2_ for 20 min in a 37 °C water bath with agitation. Digested tissues were washed with phosphate-buffered saline (PBS) and filtered through a 100 μm strainer, then the filtrate was centrifuged at 500 x *g* for 5 min to pellet SVF cells and remove the floating adipocyte layer. Red blood cells were lysed with ddH_2_O then SVF cells were stained with TOPRO3 viability dye in FACS buffer (cold PBS containing 2% fetal bovine serum and 1 mM EDTA). SVF cells from wild-type C57BL6/J mice were used as unstained and fluorescence-minus-one controls in cell sorting. Live ChAT-eGFP+ singlet cells were sorted into DMEM media using a BD FACS Aria III with a 100 μm nozzle, then immediately submitted to the University of Michigan Advanced Genomics Core at ∼1000 cells/μl for processing using the Chromium Next GEM Single Cell 3′ Kit v3. FlowJo v10 was used to visualize and analyze sorting data.

### Flow cytometry

IWAT was collected from ChAT-eGFP mice housed at thermoneutrality (30 °C), room temperature (23 °C), or acute cold (4 °C for 4 h) then SVF was isolated, as above. Cells were stained with CD45-BV650 (RRID AB_2565884) to mark immune cells and TOPRO3 to mark dead cells. Cells from WT mice were used for unstained and single-stained controls. Flow cytometry was run on a BD LSR Fortessa and analyzed in FlowJo v10.

### Single-cell RNA-sequencing and data quality control

Paired end sequencing was performed on the Illumina NovaSeq S4. Raw data were aligned to the mm10 reference mouse transcriptome using STAR and underwent pre-processing in Cell Ranger v3.0.0 (10x Genomics). Aligned pre-processed data were read from Cell Ranger into Seurat (v4.2.0) using the *CreateSeuratObject* function, with removal of cells containing less than 100 genes (*min.features = 100*) and removal of genes found in less than 10 cells (*min.cells = 10*). Low quality, dying or doublet cells were defined as having less than 200 or greater than 5000 genes or having greater than 7.5% of genes derived from mitochondrial transcripts. These cells were filtered out during pre-processing. Cell cycle regression was not performed. An elbow plot was used to determine the optimal number of principal components for post-processing (*dims = 1:25*). Semi-supervised clustering was performed using a resolution of 0.1, then non-linear dimensionality reduction was undertaken *via* Uniform Manifold Approximation and Projection (UMAP). The *FindAllMarkers* function was used to determine the cell type identity of clusters based on *log2FC*, *pct.1/pct.2* ratio and by employing the Cluster Identity Predictor (CIPR) tool with then ImmGen Mouse reference dataset of FACS-sorted murine cell types subjected to RNA-sequencing (16). Violin plots and gene feature plots were generated with the Seurat functions *VlnPlot()* and *FeaturePlot()*, respectively. For assessment of *Chat* and *Adrb2* levels, violin plots were generated using only cells with non-zero *Chat* expression.

### Cell cycle phase determination

A curated list of S phase and G2/M phase genes were provided within Seurat. To determine which phase a cell was in, the *CellCycleScoring* function was used then visualized by UMAP.

### Gene module scoring

To assess aggregate normalized gene expression, gene modules were created then plotted using the *AddModuleScore* function. For acetylcholine signaling, genes were derived from Knights *et al.* EMBO J 2021 (7): *Chat, Ache, Slc18a3, Adora2a, Chdh, Chka*.

### Analysis of macrophages

The macrophage cluster (4) was computationally subset and re-clustered *via* UMAP using 25 dimensions and a resolution of 0.3. A small number of contaminating B and T cells were removed from the resulting object. The *FindAllMarkers* function was used to determine the cell subtype identity of macrophages based on *log2FC*, *pct.1/pct.2* ratio, and related to published literature. *FindMarkers* was used to identify significantly differentially expressed genes (DEGs) between conditions, and the output of these DEGs (the top 100 up and down DEGs as ranked by *padj*) served as input for biological pathway analysis using Gene Ontology-Biological Processes, Gene Ontology-Molecular Function, and Reactome, with PantherDB’s statistical enrichment testing, with *p* < 0.05 considered significant. For Monocle trajectory gene modules, the statistical overrepresentation test was used. For pathway enrichment analyses, the annotated *Mus musculus* genome in PantherDB served as the background list of genes. Categorization of pathway terms into broad biological themes was done manually. Bubble plots of enriched GO terms were made using ggplot2.

### Trajectory analysis

For trajectory analysis, the macrophage Seurat.rds object was converted to a Monocle3.cds object using *SeuratWrappers* and its dependencies. Macrophages were re-clustered in Monocle using *cluster_cells* with *set.seed(3)*, resolution 2e-3, and K = 12 to yield six clusters. Clusters were visualized using the *plot_cells* function. Semi-supervised trajectory analysis was performed using the *learn_graph* function with branch length set to one and ncenter set to 50. For semi-supervised pseudotime analysis, the monocyte cluster was set as the origin and *order_cells* function used. To study the monocyte to infiltrating macrophage subtrajectory, the *choose_graph_segments* function was used. Modules of co-varying genes were determined using the *traj_gene_module* then visualized as a heatmap using the *pheatmap* function. Expression of individual or modules of genes, or nCount_RNA, were visualized as feature plots using the *plot_cells* function. Pseudotime regression plots were generated by extracting pseudotime values from the trajectory to create an expression data frame, then plotted with ggplot2.

### Statistics and reproducibility

For scRNAseq, for each condition, two biological replicates were used, each comprised of cells from four pooled mice. This approach was chosen to maximize biological representation and variation while retaining statistical power from high cell numbers (61). For all bioinformatics analyses, default statistical testing was used through the Seurat and Monocle3 packages, including adjustment for false discovery rate (*FindMarkers, FindAllMarkers*). For statistical comparison of pairwise gene expression and normalized aggregate gene expression (gene modules), Wilcoxon rank sum testing was performed with continuity correction. For pathway analyses (Gene Ontology and Reactome), a cutoff of *padj*<0.05 was used for DEG inputs, and ranking of term enrichment was based on RichRatio (number of DEGs found in a term divided by the total number of genes in a term) and *p* value. For all functions requiring determination of parameter settings (for example, number of dimensions or resolution for clustering), iterative parameter sweeps were performed to ensure result robustness across a range of settings. For flow cytometry analysis, normality was determined by Shapiro-Wilk testing, then one-way Analysis of Variance (ANOVA) was used to compare conditions followed by Tukey’s *post hoc* testing.

## Data availability

All scRNAseq data has been deposited to the NCBI Gene Expression Omnibus (GEO) for public accessibility under the accession “GSE303687”.

## Supporting information

This article contains supporting information.

## Conflict of interest

The authors declare that they have no conflicts of interest with the contents of this article.
