# Supplementary material for: Transcriptomic plasticity of cholinergic adipose macrophages in the acute thermogenic response
Source: J Biol Chem. 2025 Nov 7;301(12):110925. doi: 10.1016/j.jbc.2025.110925 (PMC12719665; doi:10.1016/j.jbc.2025.110925)
Supplement: Supporting Figures [file mmc1.docx]

**SUPPORTING INFORMATION**

***Transcriptomic plasticity of cholinergic adipose macrophages in the acute thermogenic response***

Alexander J. Knights, Evan J. Kim, Shanshan Liu & Jun Wu

**FIGURES**

Fig. S1: Identification of ChAT-expressing cell types by gene signature. Related to Fig. 1.

Fig. S2: Cholinergic adipose macrophages are responsive to acute cold challenge. Related to Fig. 2.

Fig. S3: Cholinergic adipose macrophages exhibit heterogeneous gene signatures. Related to Fig. 3.

Fig. S4: Origins and differentiation of cholinergic adipose macrophages. Related to Fig. 4.

**TABLES**

Table S1. FindAllMarkers output for all ChAT-eGFP+ cells.

Table S2. Macrophage DEGs.

Table S3. Trajectory gene modules.

Table S4. Trajectory gene module GO analysis.


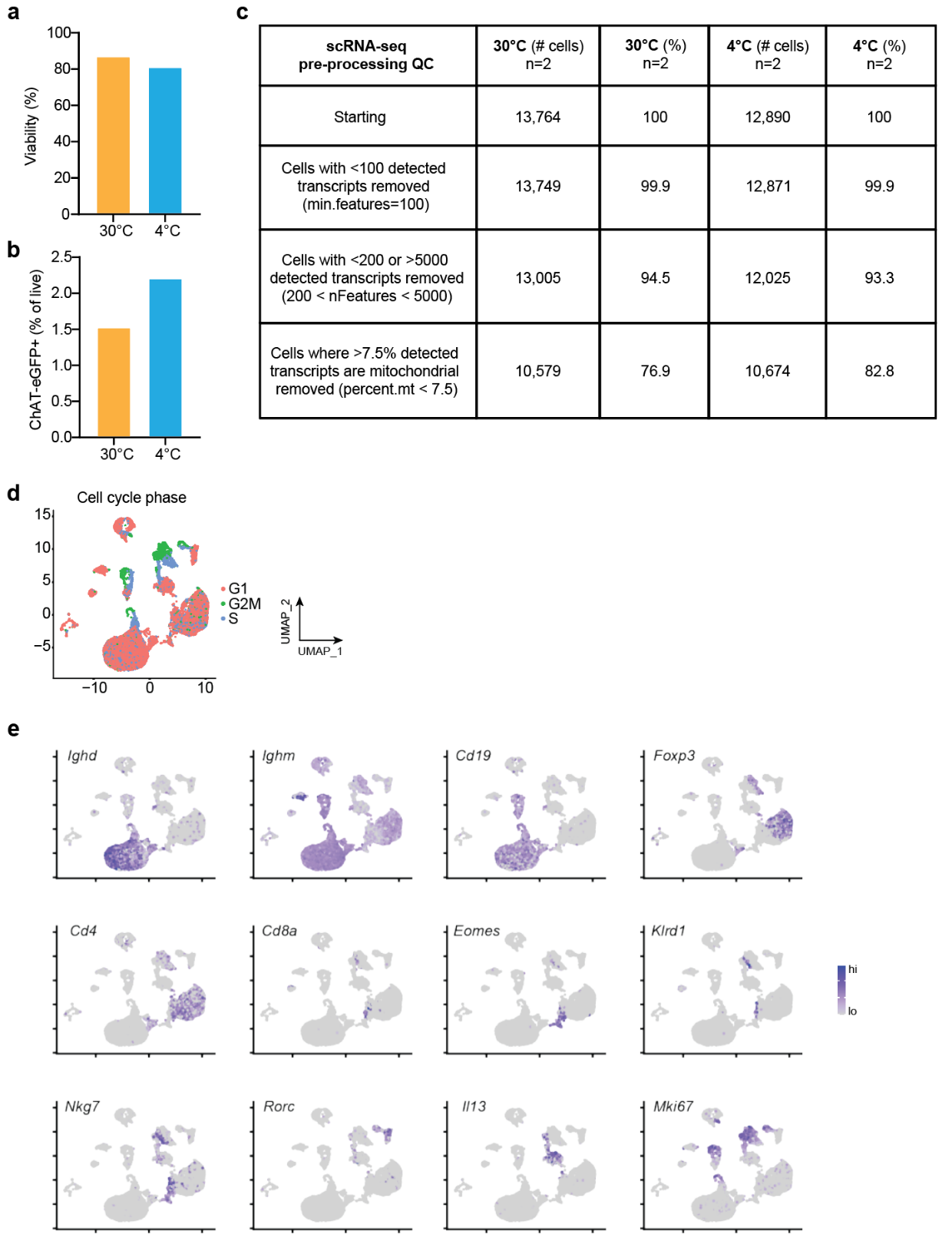


**Fig. S1: Identification of ChAT-expressing cell types by gene signature. Related to Fig. 1.** **a** Viability of cells from 30^o^C and 4^o^C conditions, measured by gating on the absence of TOPRO3 signal. **b** ChAT-eGFP+ cell percentage of all live cells in both conditions. **c** Quality control steps and the total and proportional loss of cells after each filtering step. **d** Cell cycle phase analysis showing the majority of cells in G1 phase (pink) and cycling/proliferating cells in the S (blue) or G2M (green) phase. **e** Feature plots of genes illustrating cell type subsets within clusters.


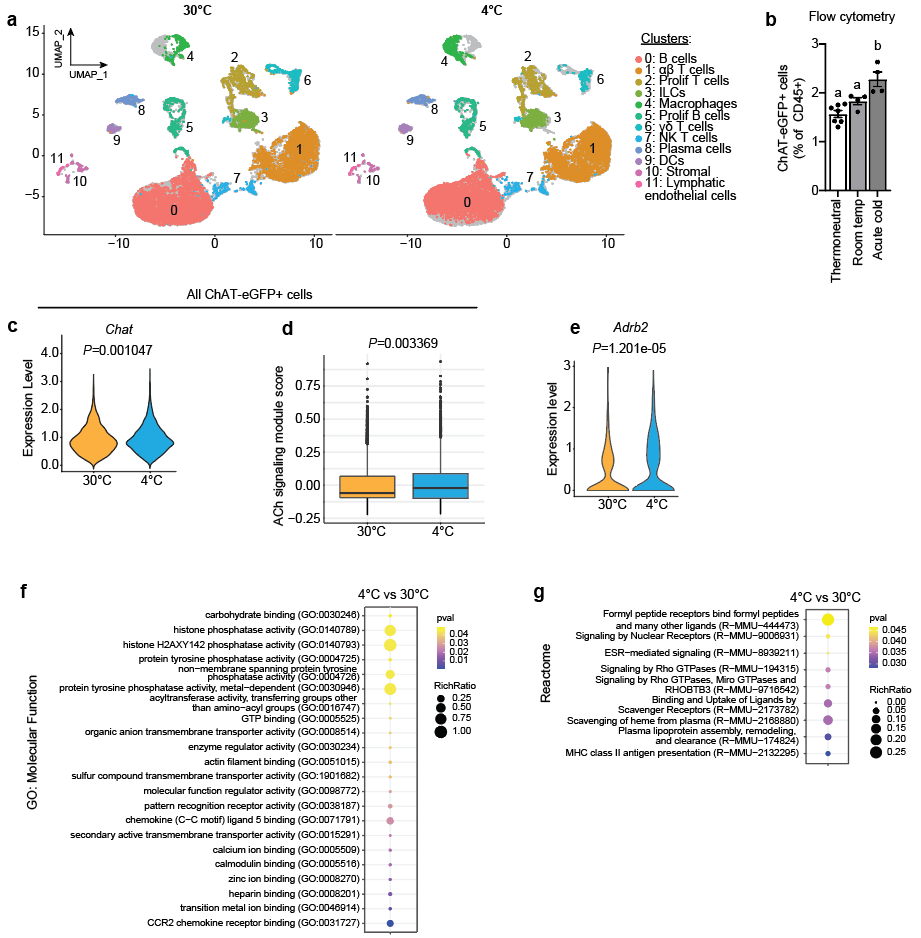


**Fig. S2: Cholinergic adipose macrophages are responsive to acute cold challenge. Related to Fig. 2.** **a** Side-by-side UMAPs showing all ChAT-eGFP+ cells from 30^o^C (left) or 4^o^C (right). **b** Flow cytometry analysis of ChAT-eGFP+ immune (CD45+) cells in IWAT from mice housed at thermoneutrality (30^o^C), room temperature (23^o^C), or acute cold exposure (4^o^C). One-way ANOVA with post-hoc Tukey testing was performed after normality testing by Shapiro-Wilk, and significantly different means are denoted by letters a or b. Individual data points represent separate mice (n=4-7). **c** Violin plot showing expression of *Chat* in all cells at 30^o^C and 4^o^C. *P* value is shown and was calculated using the Wilcoxon rank sum test. **d** Box-and-whisker plot showing normalized expression of an acetylcholine (ACh) signaling gene module in all cells at 30^o^C and 4^o^C. *P* value was calculated using the Wilcoxon rank sum test. **e** Violin plot showing expression of *Adrb2* in ChAT-eGFP+ macrophages from 30^o^C or 4^o^C. *P* value is shown and was calculated using the Wilcoxon rank sum test. **f-g** Bubble plot of GO: molecular function (MF) (**f**) and Reactome (**g**) pathways enriched in macrophages from 4^o^C. The top 100 up and down DEGs from Fig. 2d were used as input (ranked by *padj*). RichRatio is a ratio of the number of DEGs found in a pathway term over the total number of genes in that term, and pval indicates significance using the statistical enrichment test in PantherDB.


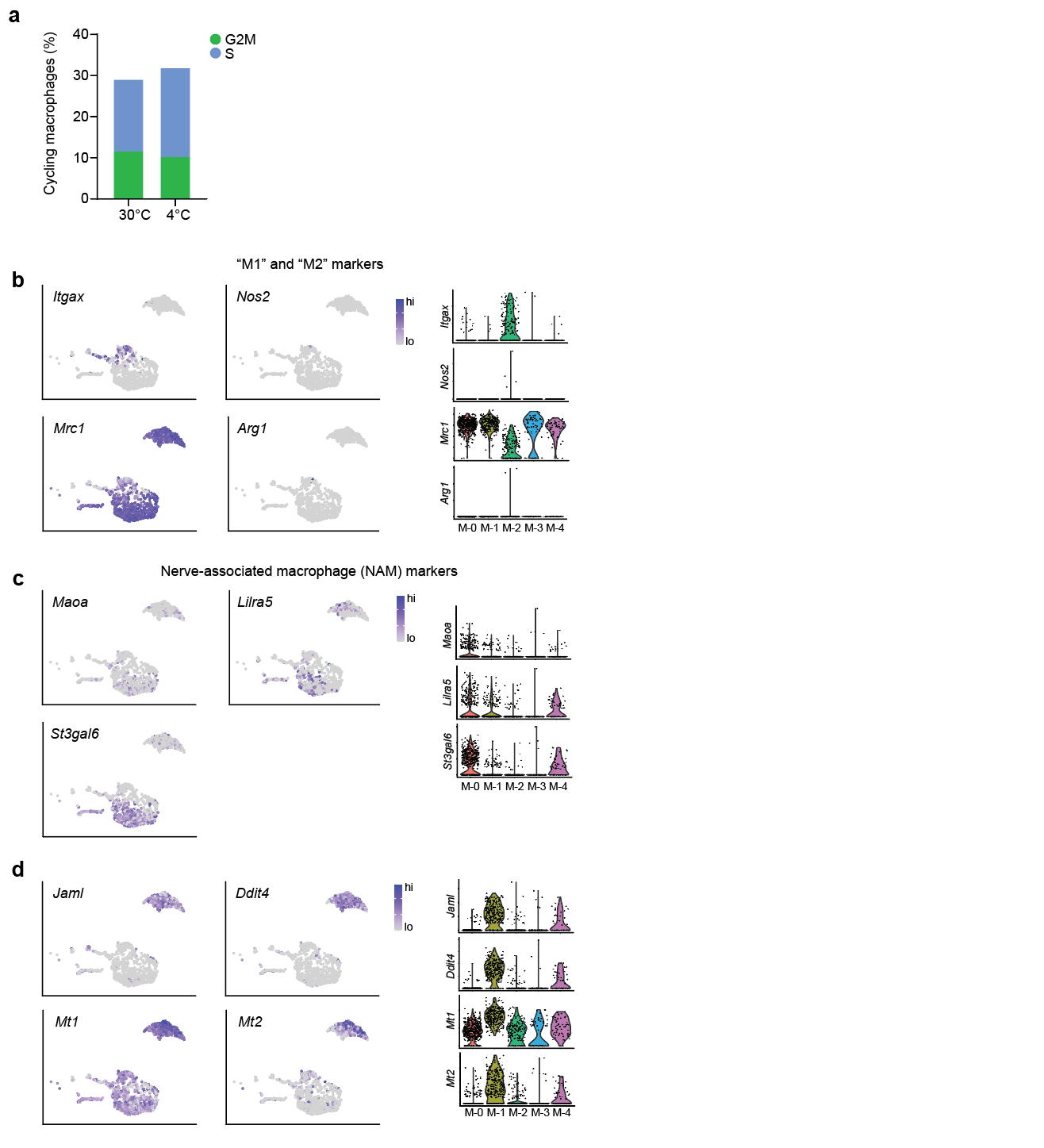


**Fig. S3: Cholinergic adipose macrophages exhibit heterogeneous gene signatures. Related to Fig. 3.** **a** G2M and S phase proliferating cells as a proportion of total ChAT-eGFP+ macrophages. **b-d** Feature (left) or violin (right) plots showing expression of “M1” and “M2” marker genes (**b**), nerve-associated macrophage (NAM) genes (**c**), and genes enriched in cluster M-1 (**d**).

**
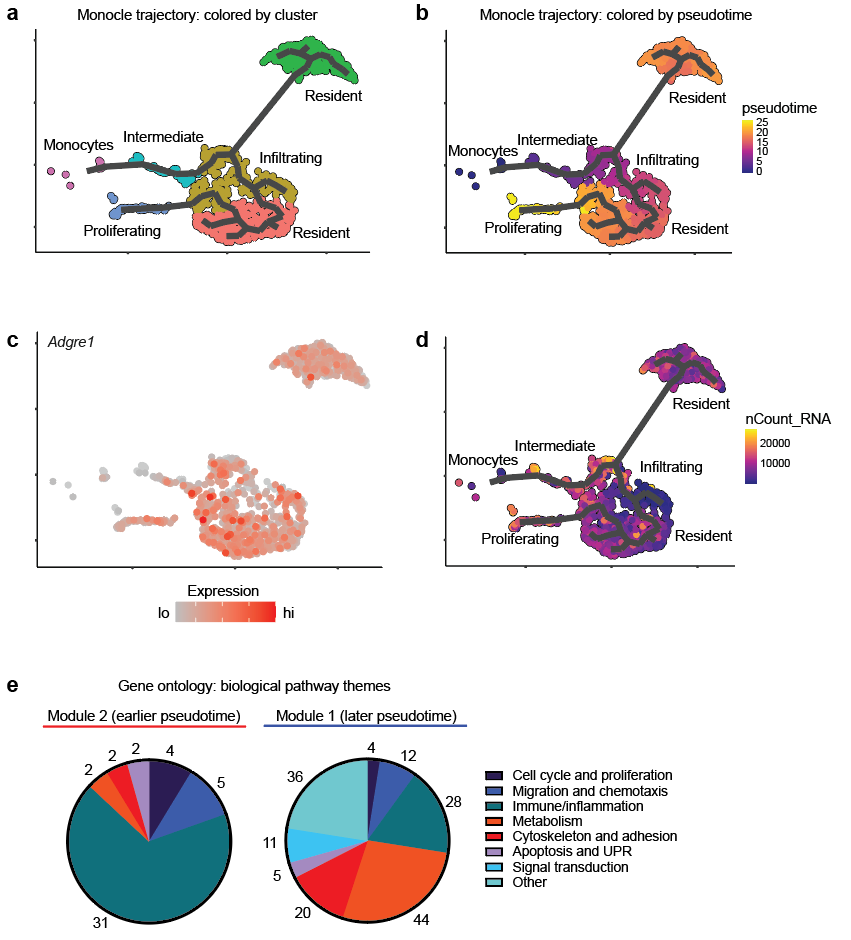
**

**Fig. S4: Origins and differentiation of cholinergic adipose macrophages. Related to Fig. 4.** **a** Trajectory analysis of ChAT-eGFP+ macrophages showing the trajectory trailmap across all clusters and partitions. **b** The trajectory trailmap colored by pseudotime. **c** *Adgre1* (F4/80) expression in ChAT-eGFP+ macrophages. **d** The total number of gene transcripts (nCount_RNA) in ChAT-eGFP+ macrophages with the trajectory trailmap overlaid. **e** Genes from modules 1 (right) and 2 (left) of Monocle trajectory analysis were subjected to statistical overrepresentation testing of enriched biological pathways across pseudotime. The broad themes of enriched pathways in each module are shown, with the number of GO terms shown next to each pie slice.
